# Acoustic Bubble Sensing Techniques and Bioapplications

**DOI:** 10.3390/bios16020088

**Published:** 2026-01-31

**Authors:** Renjie Ning, Jonathan Faulkner, Mengren Wu, Yuan Gao

**Affiliations:** 1 Department of Mechanical Engineering, University of Memphis, Memphis, TN 38152, USAjflkner3@memphis.edu (J.F.); 2 Department of Mechanical Engineering, Stanford University, Stanford, CA 94305, USA

**Keywords:** acoustic bubbles, oscillating bubbles, biosensing, biomechanical sensing, bioapplications

## Abstract

Acoustic bubbles are emerging as powerful microscale sensors that convert local biochemical and biomechanical cues into measurable signals in a remote, label-free, and clinically compatible manner. Originally developed as vascular contrast agents, microbubbles are now engineered so that their resonance frequency, nonlinear oscillations, cavitation emissions, microstreaming, and radiation-force-induced motion encode information about pressure, rheology, oxygenation, and cell or tissue mechanics. In this review, we first summarize the fundamental physics of bubble dynamics, and then describe how these dynamics are translated into practical sensing observables. We then highlight key bioapplications where acoustic bubbles function as environment-responsive probes, ranging from hemodynamic pressure and fluid rheology to oxygen levels and cellular mechanics. Across these examples, we emphasize advantages such as non-invasive and wireless readout, high sensitivity arising from nonlinear bubble dynamics, and biochemical and molecular tunability. Finally, we outline current challenges and future opportunities for translating acoustic bubble-based sensing into robust, quantitative tools for biomedical applications.

## 1. Introduction

Quantitative sensing of the biochemical and biophysical microenvironment is important for biomedical research and clinical practice. Parameters such as pressure, flow, viscosity, oxygenation, pH, and tissue stiffness, which regulate vascular function, tissue homeostasis, immune response, and tumor progression, are closely associated with cardiovascular disease, cancer, fibrosis, and neurological disorders [1,2,3]. Microphysiological systems also require sensing modalities that can operate robustly under defined flow, shear, and culture conditions [4]. Therefore, reliable sensing techniques are essential for disease diagnosis and for the development of in vitro microphysiological systems. Conventional optical, electrochemical, or label-based sensors often require direct contact, line-of-sight access, or exogenous labels, which can perturb the systems they are meant to measure [5,6]. There is thus a growing need for minimally invasive, remote sensing strategies that can report on local microenvironmental conditions in real time and in situ.

Gas-filled microbubbles have long been used as ultrasound contrast agents for vascular imaging, because they are highly compressible and have a large acoustic impedance mismatch with surrounding tissues, which makes them efficient scatterers of ultrasound [7,8]. In the biomedical field, microbubble formulations typically consist of micron-scale gas cores encapsulated by lipid, protein, or polymer shells that stabilize the bubble and allow functionalization with targeting ligands [9,10]. Under diagnostic acoustic pressures, these microbubbles undergo radial oscillations that are orders of magnitude larger than those of tissue, leading to strong, nonlinear backscatter and characteristic harmonic signatures [11]. Over the past two decades, this unique acoustic response has been exploited not only for improved imaging contrast, but also for targeted delivery, sonoporation, and microfluidic actuation [12,13,14]. In parallel with these developments, there is growing interest in engineering microbubbles as active, environment-responsive probes for sensing, rather than using them only as passive contrast carriers.

Within this context, acoustic bubble sensing refers to using bubble vibrations under ultrasound, and the resulting sound and fluid motion, to convert local microenvironmental conditions into measurable signals. Because the resonance frequency, damping, and nonlinear response of microbubbles are exquisitely sensitive to ambient pressure, fluid rheology, shell mechanics, and nearby boundaries, they respond strongly to subtle changes in the biochemical or mechanical environment. Acoustic bubble sensing offers several advantages in bioapplications. First, it is remote and wireless: microbubbles can be interrogated by external ultrasound transducers without physical contact or optical access, enabling sensing in deep tissues, enclosed microchannels, and organ-on-a-chip systems [15,16]. Second, the technique is multifunctional: the same microbubble can simultaneously serve as an imaging contrast agent, a local actuator (via radiation force or cavitation), and a sensor whose emissions or motion encode environmental parameters [17,18]. Third, acoustic sensing is highly sensitive and nonlinear: small perturbations in pressure, viscosity, shell stiffness, or gas composition can produce large, measurable changes in resonance frequency, harmonic content, or subharmonic amplitude [19,20,21,22]. Fourth, microbubbles are chemically and biologically tunable: shell chemistry and gas payload can be engineered to respond to specific analytes such as oxygen, nitric oxide, or biomolecular binding events, coupling biochemical processes directly to mechanical and acoustic signatures [23]. Finally, acoustic bubble sensing is biocompatible and minimally invasive when operated within diagnostic ultrasound exposure limits, reducing the risk of contamination or perturbation compared with implanted sensors [24,25].

Despite rapid growth in the area of acoustic bubble sensing, many excellent reviews on microbubbles focus on their roles as contrast agents for imaging or as cavitation nuclei for drug and gene delivery [26,27,28,29]. The acoustic bubble sensing—linking fundamental oscillation physics to sensing modalities and bioapplications—was not reported. This review aims to summarize the underlying physics of bubble oscillation and how these dynamics are transduced into measurable signals. In addition, we also highlight the bioapplications of these techniques and discuss the limitations and perspectives of acoustic bubble sensing.

## 2. Acoustic Bubble Sensing Mechanisms

Acoustic bubble-based sensing relies on the intimate coupling between bubble oscillations and their surrounding microenvironment. When driven by ultrasound, a gas bubble responds as a damped resonator whose dynamics are governed by the balance of gas pressure, surface tension, viscous stresses, and external loading from the acoustic field and nearby boundaries [30,31]. Small changes in ambient pressure, fluid rheology, shell properties, or biochemical composition of the gas and shell can therefore produce measurable changes in bubble radius–time dynamics and in the associated acoustic and hydrodynamic fields. In this section, we first review bubble oscillations and then discuss how these oscillations are transduced into measurable signals.

### 2.1. Bubble Oscillations and Biochemical Modulation

#### 2.1.1. Radial Dynamics and Linear Resonance

The radial dynamics of a single spherical bubble of equilibrium radius $R_{0}$ in a Newtonian liquid of density ρ and viscosity μ are often described by the Rayleigh–Plesset equation [30,31,32]:

(1) $$\rho \left(R\ddot{R}+\frac{3}{2}{\dot{R}}^{2}\right)=p_{g}\left(R,t\right)-p_{0}-p_{\mathrm{ac}}\left(t\right)-\frac{2\sigma }{R}-4\mu \frac{\dot{R}}{R},$$

where *R* is the bubble radius, $\dot{R}$ and $\ddot{R}$ represent the first- and second-order time derivatives of the bubble radius (∂R/∂t and $\partial^{2}R/\partial t^{2}$, respectively), $p_{g}\left(R,t\right)$ is the gas pressure inside the bubble, $p_{0}$ is the ambient pressure, $p_{\mathrm{ac}}\left(t\right)$ is the driving acoustic pressure, and σ is the surface tension.

Although the Rayleigh–Plesset equation assumes a Newtonian liquid, biological samples (e.g., blood, plasma, protein solutions) exhibit non-Newtonian rheology, characterized by shear-thinning or viscoelastic behavior. However, the validity of the Newtonian approximation hinges on the local shear rate at the bubble wall, approximated by $|\dot{R}/R|\approx 2\pi f\left(R_{\epsilon }/R_{0}\right)$, where *f* is the driving frequency and $R_{\epsilon }$ is the oscillation amplitude [33]. For ultrasonic sensing applications (f≳20 kHz), the rapid motion of the bubble wall generates high shear rates, typically on the order of 10^4^–10^6^ s^−1^ for micron-sized bubbles [34]. Rheological studies of biological fluids demonstrate that at these high strain rates, the fluid microstructure is disrupted and the viscosity reaches a constant plateau ($\mu_{\infty }$) [35,36]. Consequently, within this high-frequency regime, the medium effectively behaves as a Newtonian fluid [37].

**Figure 1:**
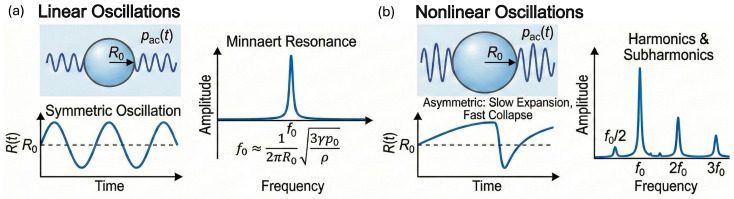
Regimes of acoustic bubble oscillation. $R_{0}$ is the equilibrium radius of a single bubble. (**a**) The linear oscillation regime occurring at low acoustic pressure amplitudes ($p_{ac}$). (**b**) Nonlinear oscillation regime where increased acoustic pressure ($p_{ac}$) induces stable cavitation, characterized by the generation of harmonics ($2f_{0},3f_{0}$) and subharmonics ($f_{0}/2$), and inertial cavitation.

Under this assumption, for small-amplitude oscillations about equilibrium ($R\left(t\right)=R_{0}+r\left(t\right)$, with $\left|r\right|\ll R_{0}$) and an isentropic gas law ($p_{g}R^{3\gamma }=\mathrm{const}.$), the system simplifies to a harmonic oscillator with a natural (Minnaert) resonance frequency (Figure 1a) [38]:

(2) $$f_{0}=\frac{1}{2\pi R_{0}}\sqrt{\frac{3\gamma p_{0}}{\rho }},$$

where γ is the polytropic exponent of the gas. Equation (2) highlights that even modest changes in ambient pressure $p_{0}$, gas composition (which affects γ), or effective density ρ can shift the bubble resonance frequency, a property that underpins many pressure- and oxygen-sensing schemes [39,40].

Viscous and thermal damping broaden the resonance peak and reduce oscillation amplitude [31,41]. In biological and microenvironments, additional damping arises from nearby boundaries (such as vessel or channel walls) and from the viscoelastic response of surrounding tissues. These effects alter both the effective resonance frequency and the quality factor (*Q*) of the bubble, which describes how sharply and strongly the bubble resonates before its oscillations decay. Models incorporating these boundary effects are essential for interpreting microbubble sensing in confined microvessels, organ-on-chip channels, and tissue-like gels [42,43].

#### 2.1.2. Nonlinear Oscillations and Cavitation Regimes

At modest acoustic pressures, microbubble oscillations remain approximately linear, and the scattered field is dominated by the fundamental frequency. As the driving pressure increases, the Rayleigh–Plesset equation becomes strongly nonlinear, giving rise to harmonic, ultraharmonic, and subharmonic responses as well as large-amplitude radial excursions (Figure 1b) [44]. The subharmonic response, in particular, is highly sensitive to ambient pressure and shell properties and has been widely exploited for subharmonic-aided pressure estimation (SHAPE) [45,46,47].

At higher pressures, inertial cavitation can occur, characterized by rapid bubble growth and violent collapse that produces broadband acoustic emissions and potentially mechanical damage [30,48,49,50]. Stable cavitation typically refers to sustained, large-amplitude oscillations with pronounced harmonic and ultraharmonic content but without catastrophic collapse [51,52]. For sensing applications, stable, controlled nonlinear oscillations are generally desirable because they provide enhanced sensitivity to environmental parameters while minimizing tissue damage, whereas inertial cavitation is used more cautiously, for example when cavitation dose itself is the sensing metric for barrier opening or biofilm disruption [53].

#### 2.1.3. Encapsulated Contrast Agents and Shell Rheology

Ultrasound contrast agents and many sensing microbubbles are encapsulated by shells composed of lipids, proteins, or polymers, which introduce additional elasticity and viscosity. Several modified Rayleigh–Plesset models have been developed to account for the shell, including the Church model [54], the Marmottant model [21], and viscoelastic encapsulation models [55,56]. In a simplified form, the shell adds an elastic term and a viscous term to Equation (1),

(3) $$p_{g}\left(R,t\right)-p_{0}-p_{\mathrm{ac}}\left(t\right)=\rho \left(R\ddot{R}+\frac{3}{2}{\dot{R}}^{2}\right)+\frac{2\sigma_{\mathrm{eff}}\left(R\right)}{R}+4\mu \frac{\dot{R}}{R}+\frac{4\kappa_{s}}{R}\dot{R},$$

where $\sigma_{\mathrm{eff}}\left(R\right)$ is an effective (possibly nonlinear) surface tension and $\kappa_{s}$ is the surface (shell) viscosity. Shell elasticity stiffens the bubble, increasing $f_{0}$, while shell viscosity provides additional damping that broadens the resonance peak and suppresses large nonlinear excursions [57,58].

Shell rheology is sensitive to biochemical composition, temperature, ligand density, and gas exchange. High-speed imaging and single-bubble spectroscopy have been used to extract shell elasticity and viscosity from measured radius–time curves and scattered pressure, providing a quantitative link between shell composition and acoustic response [57,59]. The encapsulated bubbles can be engineered with tailored mechanical properties and that small changes in shell organization produce measurable shifts in resonance and damping, which is a key mechanism for biochemical sensing.

#### 2.1.4. Size-Dependent Acoustics and Population Effects

The sensing performance of acoustic bubbles is intrinsically governed by their equilibrium size, which dictates both the operating frequency and the dominant damping mechanisms. From the Minnaert approximation, the resonant frequency scales inversely with radius ($f_{0}\propto R_{0}^{-1}$), creating distinct operational regimes [38]. Nanobubbles (<1 μm) resonate at high frequencies (>15 MHz) and are dominated by shell viscosity, making them suitable for extravascular imaging where capillary permeability is required [60]. The micron regime (1–8 μm) represents the clinical standard, resonating within the diagnostic window (1–10 MHz) with strong nonlinear scattering ideal for vascular perfusion sensing. Bubbles exceeding the capillary filtration threshold (>8 μm) resonate at sub-megahertz frequencies (<1 MHz). These bubbles are dominated by radiation damping rather than shell viscosity. Their large geometric cross-section enables them to undergo violent inertial collapse at lower acoustic pressures compared to smaller bubbles, making them potent agents for mechanically focused sensing and actuation in microfluidic channels or industrial flows [51].

The precision of the sensing regimes is often constrained by population polydispersity, where broad size distributions result in a ‘spectral smearing’ effect where the overlapping responses of individual bubbles flatten the collective resonance peak [61]. To maximize sensitivity, recent advances focus on the production of monodisperse populations, such as T-junction generators and flow focusing devices, which yield sharp, high-quality resonance signatures and enable more quantitative acoustic sensing [62,63,64]. Furthermore, integrated microfluidic trapping strategies allow for the precise spatial localization of these calibrated bubbles, ensuring consistent interrogation conditions and minimizing variability due to flow or buoyancy [65].

#### 2.1.5. Biochemical Modulation of Bubble Mechanics

For sensing applications, the internal gas and shell can be functionalized to transduce specific biochemical events into mechanical changes. For example, hemoglobin-coated microbubbles (HbMBs) have been applied as an oxygen sensor. Oxygen binding alters hemoglobin conformation and compressibility, which can effectively modulate shell stiffness and thereby shift the resonance frequency and harmonic structure [23,66,67]. Similarly, attachment of ligands, antibodies, or glycoconjugates to the shell can change packing density, interfacial viscoelasticity, and surface tension, tuning bubble dynamics in response to receptor binding or enzymatic cleavage [68].

Biochemical processes that alter gas composition (e.g., O_2_/CO_2_ exchange, NO loading and release) also modify the effective gas density and polytropic behavior, which can influence resonance and damping [69]. At microscale, such biochemical modulations are superimposed on changes in ambient pressure, viscosity, and boundary conditions. Therefore, sensing strategies exploit the combined sensitivity of encapsulated bubbles to both mechanical and biochemical cues, reading out information via changes in the acoustic and hydrodynamic fields.

### 2.2. From Bubble Oscillations to Measurable Signals

While bubble dynamics occur at the micron scale, they generate acoustic, hydrodynamic, and mechanical signatures that are readily accessible to external measurement. In this section, we outline how oscillations are transduced into measurable quantities used for sensing: backscatter and attenuation, spectral features and cavitation emissions, microstreaming and advective transport, and radiation-force-induced displacement linked to mechanotransduction.

#### 2.2.1. Backscatter and Attenuation

The primary measurable consequence of bubble oscillations is the scattered acoustic field. Based on the unified theoretical framework by Hilgenfeldt et al., the scattered pressure is composed of two contributions: “active” emission, driven by the volumetric acceleration of the bubble, and “passive” emission, arising from the bubble’s presence as an obstacle. For gas bubbles, active emission is dominant [70]. While the scattering cross-section $\sigma_{sc}$ is often cited as increasing with the sixth power of the radius ($R_{0}^{6}$) near resonance, this scaling is strictly valid only in the small-amplitude, long-wavelength linear limit [42,70]. A comprehensive model must account for the transition to the strongly nonlinear regimes typical of diagnostic ultrasound. Under these conditions, the violent collapse of the bubble leads to extraordinary wall accelerations, causing active sound emission to exceed linear predictions by several orders of magnitude. Consequently, the energy balance shifts: in the linear regime, viscous absorption dominates ($\sigma_{abs}\gg \sigma_{sc}$). However, as driving pressure increases, the system transitions toward a scattering-dominated regime ($\sigma_{sc}\gg \sigma_{abs}$), converting the majority of incident energy into re-radiated sound.

For sensing applications, these distinct acoustic regimes are exploited to probe specific environmental parameters. High-amplitude active backscatter is utilized for pressure sensing, such as in Subharmonic-aided Pressure Estimation (SHAPE), where pressure-dependent subharmonic amplitudes provide a high-sensitivity marker [46]. Conversely, the attenuation spectrum serves as a sensitive probe for the local rheological environment. Changes in fluid viscosity—associated with tissue stiffening or fibrosis—enhance the viscous damping term, which broadens the resonance peak and reduces the peak backscatter amplitude [71,72]. By utilizing these spectral signatures, bubble–environment interactions allow for the non-invasive monitoring of metabolic states and pathological transitions.

#### 2.2.2. Spectral Analysis and Cavitation Monitoring

The nonlinear nature of bubble oscillations produces a rich frequency spectrum containing the fundamental, harmonics ($2f_{0},3f_{0},\ldots$), ultraharmonics, and subharmonics ($f_{0}/2$), as well as broadband noise under inertial cavitation. Spectral analysis of these components provides multiple independent channels for sensing. The position and amplitude of the fundamental resonance peak can be used to infer bubble size, ambient pressure, and shell stiffness.

Passive cavitation detection (PCD) and spectral monitoring of broadband emissions enable quantification of cavitation dose and classification of the cavitation regime. For example, in vascular-permeability studies, correlations between specific spectral features (e.g., stable cavitation dose, ultraharmonic energy) and physiological readouts (e.g., transendothelial electrical resistance (TEER) or dye extravasation) have been established. These correlations are used to define safe operating windows and establish acoustic emissions as surrogate sensing metrics for barrier opening [73,74,75].

#### 2.2.3. Microstreaming and Advective Transport

**Figure 2:**
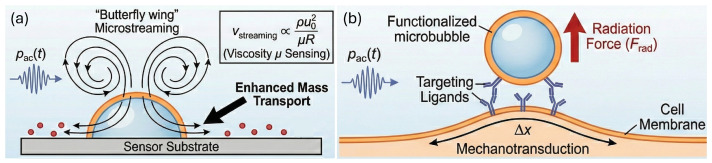
(**a**) Acoustic microstreaming generated by a bubble near a boundary, used for enhancing mass transport to a sensor surface and for sensing fluid viscosity (μ). (**b**) Acoustic radiation force ($F_{\mathrm{rad}}$) exerted on a targeted microbubble, used to apply localized mechanical strain (Δx) to biological membranes for mechanotransduction studies.

Acoustic bubbles also drive steady, vortical flows known as acoustic microstreaming, arising from nonlinear interactions in the oscillatory boundary layer (Figure 2a) [76,77]. Near a rigid wall or within a confined microchannel, these streaming flows form recirculating vortices whose velocity magnitude and spatial extent scale with the square of the oscillation amplitude and inversely with viscosity. Microstreaming enhances advective transport around the bubble, increasing local shear and mixing.

For sensing, microstreaming can be exploited in two ways. First, the streaming flow field itself encodes information about viscosity and rheology: more viscous fluids generate thicker Stokes boundary layers and weaker, more compact vortices. Second, streaming-enhanced mass transport can dramatically improve the sensitivity and response time of surface-based sensors. For example, acoustic bubbles embedded near sensing surfaces drive analytes toward capture sites, increasing effective on-rates and amplifying signal changes in optical or electrochemical measurements.

In biological contexts, microstreaming-induced shear can also be used as a local perturbation to probe cell adhesion strength, thrombus stability, or biofilm mechanical integrity, where changes in streaming patterns or debris size distributions provide functional readouts of structural weakening during therapy.

#### 2.2.4. Radiation-Force-Induced Displacement and Mechanotransduction

Time-averaged interactions between an oscillating bubble and an ultrasound field generate a primary acoustic radiation force that can displace the bubble along the beam axis. For a small, compressible scatterer in a standing or weakly focused wave, the time-averaged force $F_{\mathrm{rad}}$ scales with the scattering cross-section and the spatial gradient of acoustic energy density (Figure 2b). In practice, radiation force is widely used to translate attached or free bubbles, enabling several sensing and mechanobiology modalities.

When microbubbles are tethered to cells, radiation-force-induced displacements exert controlled mechanical loads on the cell surface while the bubble motion can be tracked optically or ultrasonically [78,79]. The amplitude and relaxation dynamics of bubble–cell displacement provide a sensitive measure of cell stiffness, cortical tension, and viscoelasticity.

Radiation-force induced bubble motion can also be monitored acoustically. For example, in radiation-force imaging of endothelialized vessels or in vessel-on-chip systems, the displacement of a bubble under known acoustic driving conditions provides information about local drag, adhesion, and barrier mechanics [80,81]. Coupling these mechanical perturbations with biochemical readouts, such as intracellular calcium, focal adhesion remodeling, or lineage-specific gene expression, links bubble motion not only to static mechanical properties, but also to mechanotransduction pathways and downstream biochemical responses [82].

## 3. Exemplary Applications of Acoustic Bubble Sensing

### 3.1. Pressure and Fluid-Rheology Sensing

Acoustic microbubbles act as highly sensitive pressure and rheology sensors because ambient pressure and fluid mechanical properties directly modify their resonance conditions. The resonance frequency of a bubble increases with rising ambient pressure or decreasing radius, whereas fluid viscosity and density influence damping, oscillation amplitude, and subharmonic generation. These dependencies have enabled a broad class of microbubble-based acoustic sensors capable of reporting hemodynamic pressure, tissue stiffness, blood rheology, clotting behavior, and other physiologically relevant mechanical parameters.

**Figure 3:**
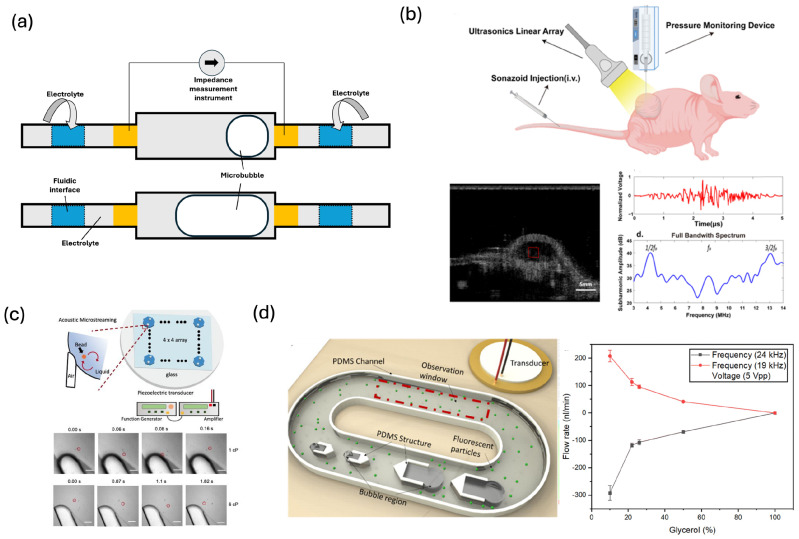
(**a**) MEMS-integrated microbubble sensor for quantifying local pressure changes [83]. (**b**) In vivo monitoring of tumor interstitial fluid pressure using subharmonic microbubble emissions. Reprinted with permission from reference [87]. (**c**) μVAST device (Microfluidic Viscometer using Acoustic Streaming Transducers) for viscosity measurement. Scale bar: 50 μm. Reprinted with permission from reference [93]. (**d**) Acoustic bubble-based bidirectional micropump for testing blood-mimicking fluids, where the flow rate of fluorescent tracer particles driven by bubble-induced streaming is measured as a function of viscosity. Reprinted with permission from reference [94].

Early studies established that the subharmonic emissions from phospholipid-coated microbubbles shift consistently with hydrostatic pressure, allowing passive pressure estimation without inducing inertial cavitation [39]. Building on this principle, Li et al. demonstrated that microbubble attenuation and resonance frequency change linearly and nonlinearly with pressure, enabling high-resolution microscale blood-pressure sensing over physiologically relevant ranges [40]. Yu et al. created an MEMS-integrated microbubble sensor that converted oscillation amplitude into electrochemical readouts, providing a miniaturized approach for quantifying pressure changes with enhanced sensitivity (Figure 3a) [83]. More recent systematic studies have optimized the excitation conditions for subharmonic-aided pressure estimation. Azami et al. mapped how drive frequency and mechanical index modulate subharmonic sensitivity, identifying operational regimes that maximize pressure responsiveness in clinical microbubbles [45]. Additional in vitro and in vivo work has extended this concept to physiological systems: subharmonic microbubble emissions have been correlated with intracardiac pressure, portal vein pressure, and tumor interstitial pressure (Figure 3b) [69,84,85,86,87]. These studies collectively position subharmonic microbubble sensing as a promising tool for noninvasive hemodynamic monitoring in cardiovascular and hepatic diseases.

Beyond pressure, acoustic microbubbles have emerged as powerful rheological probes because viscosity strongly influences oscillatory dynamics and energy dissipation. High-speed imaging studies have revealed that shell viscoelasticity significantly modulates bubble behavior. Cattaneo et al. quantified shell viscosity of lipid-coated microbubbles at the single-bubble level using high-speed videomicroscopy and nonlinear bubble-dynamics modeling, resolving longstanding inconsistencies in shell-rheology measurements and improving predictive accuracy of acoustic sensing models [88]. Many works from microbubble-interfacial rheology research have shown that surrounding-fluid viscosity suppresses inertial cavitation, changes fragmentation thresholds, increases damping, and modifies the harmonic structure of bubble oscillations [71,89,90]. These viscosity-dependent modulation patterns provide a physical foundation for microbubble-based rheology and clotting diagnostics.

Microbubble sensing has also been applied to characterize biofluid rheology relevant to coagulation, inflammation, and tissue mechanics. Studies using resonant scattering or attenuation have shown that increasing viscosity, due to fibrin formation, polymerization, or elevated plasma protein content, results in reduced oscillation amplitude and enhanced damping, allowing acoustic identification of early clotting events. Encapsulated microbubbles embedded in viscoelastic biomaterials or high-viscosity hydrogels exhibit measurable shifts in resonance and shell elasticity, enabling microbubble-based rheometry for soft biomaterials and engineered tissues [72,91].

Confinement effects in microvessels and microfluidic channels have added further richness to pressure and rheology sensing. Microbubble oscillations are altered markedly in small compliant vessels, where vessel-wall viscosity and elasticity modulate bubble dynamics. Yusefi and Helfield modeled bubble–bubble interactions inside viscoelastic microvessels and found that wall damping suppresses oscillation amplitude while bubble–bubble coupling amplifies size-dependent oscillation modes [92]. These findings highlight that microbubble-based measurements in microchannels or in vivo must account for geometric confinement and effective medium viscosity, particularly when interpreting pressure or flow-dependent microbubble behavior in organ-on-chip systems.

In addition to analyzing oscillatory signatures directly, viscosity can also be inferred from microbubble-induced acoustic streaming. Confined bubbles driven by ultrasound generate steady streaming vortices whose velocity profiles depend sensitively on the Stokes boundary-layer thickness. Jiang et al. developed the μVAST (microfluidic Viscometer using Acoustic Streaming Transducers), which uses acoustically driven microbubbles to measure viscosity by quantifying streaming velocity via micro-PIV and particle tracking (Figure 3c) [93]. More viscous fluids produce thicker boundary layers and weaker streaming fields, enabling rapid viscosity extraction using only microliter-scale samples. Related microstreaming-based devices have demonstrated quantitative viscosity detection in complex biological fluids, including blood-mimicking fluids (Figure 3d) [94], polymeric solutions and protein-rich biofluids [95], showing the potential of microbubble-driven streaming as a generalizable microscale rheometry tool.

### 3.2. Oxygen and Metabolic Sensing

**Figure 4:**
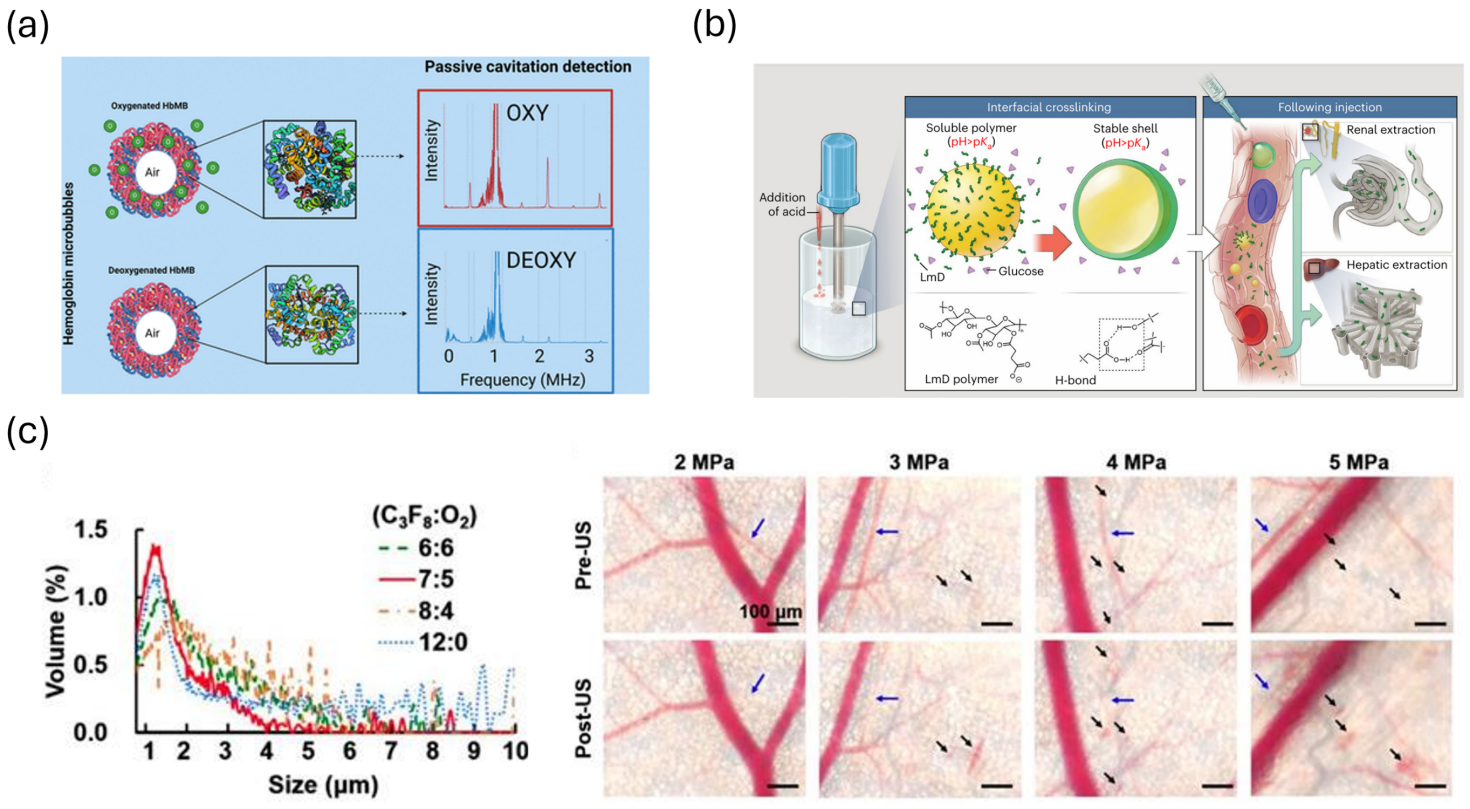
(**a**) Acoustic cavitation response of oxygenated and deoxygenated hemoglobin microbubbles. Reprinted with permission from reference [23]. (**b**) Design and characterization of rapidly dissolving oxygen microbubbles for systemic intravenous oxygen delivery. Reprinted with permission from reference [98]. (**c**) The vascular constriction (blue arrows) and disruption (black arrows) during $O_{2}$-MBs destruction under various acoustic pressures. Reprinted with permission from reference [97].

Acoustic bubbles have been applied as a uniquely powerful tool for monitoring oxygenation and metabolic state. Changes in local oxygen concentration alter the concentration gradient across the bubble shell, driving gas diffusion and changing the bubble’s size and stability over time. These shifts in dissolution kinetics and resonance can be detected in real time using diagnostic ultrasound. Hemoglobin-functionalized microbubbles (HbMBs) have been investigated for oxygen direction, which directly couples hemoglobin’s oxygen-binding behavior to bubble mechanics. Chaudhary and colleagues demonstrated that oxy- and deoxy-hemoglobin microbubbles exhibit distinct acoustic attenuation and harmonic signatures, enabling a form of “acoustic BOLD imaging” analogous to MRI-BOLD but with far higher temporal resolution and compatibility with portable clinical ultrasound systems (Figure 4a) [23]. Rastegar et al. expanded the platform by engineering PEGylated HbMBs with improved stability and demonstrated sustained oxygen responsiveness under physiological conditions, a key step toward in vivo deployment [67]. Pathour et al. took the field further by applying deep neural networks to raw RF ultrasound data, showing that machine-learning models can classify oxygenation states of HbMBs with striking accuracy, even when the underlying oscillation differences are small [96].

A second direction is to use oxygen-loaded microbubbles (O_2_MBs) as both oxygen carriers and indirect reporters of metabolic state. In these systems, ultrasound serves primarily to trigger or modulate oxygen release, while changes in oxygen tension are quantified using a combination of imaging and invasive or optical measurements. Eisenbrey et al. developed an ultrasound-sensitive oxygen microbubble formulation (SE61_*O*_2__) and showed that ultrasound-triggered destruction of these bubbles significantly increased dissolved oxygen in vitro and elevated intratumoral pO_2_ in mouse breast tumors, with contrast-enhanced ultrasound confirming microbubble delivery [69]. Ho et al. used oxygen microbubbles with ultrasound to normalize tumor vasculature, quantifying a transient window of improved perfusion and reduced hypoxia via contrast-enhanced ultrasound time–intensity curves and histological hypoxia markers (Figure 4c) [97]. The recent work by Mancebo et al. represents a significant advancement: they engineered pH-responsive, rapidly dissolving polymeric oxygen microbubbles and used ultrasound imaging to track their acoustic dissolution kinetics in vivo, demonstrating safe intravenous delivery of high oxygen payloads that improves arterial oxygenation, survival, and organ protection in a swine model of severe hypoxaemia (Figure 4b) [98].

Microfluidic and organ-on-chip models provide additional insight into how oxygen-delivering bubbles and capsules behave under physiologically relevant flows and confined geometries [99]. In this context, microfluidic bubble generation technologies are valuable primarily because they provide reproducible control over bubble size, shell composition, and gas loading, ensuring well-defined resonance and nonlinear responses that can be quantitatively linked to oxygen tension [65,100,101]. Flow-focusing and T-junction devices have been used to create monodisperse microbubbles and oxygen carriers with tightly controlled mechanical properties, providing consistent oscillatory behavior ideal for sensing [102,103,104,105]. Confinement within micron-scale channels and tissue-mimicking chambers alters the local acoustic field, viscous boundary layers, and bubble–wall interactions. These changes modulate resonance frequency, damping, and subharmonic output, which need to be accounted for when extracting oxygen or metabolic information from acoustic signals.

Moreover, oscillating microbubbles in microchannels generate strong acoustic microstreaming flows that enhance convective transport of oxygen and metabolites to and from sensor surfaces or cells. De Vellis and co-workers demonstrated that resonantly driven microbubbles trapped in microcavities can dramatically accelerate analyte transport to surface-based sensors, reducing response times by over an order of magnitude [106]. This coupling between streaming-enhanced mass transport and biochemical readout is particularly attractive for microscale oxygen and metabolite sensing, where diffusion limits are otherwise severe.

### 3.3. Cellular Mechanical Phenotyping

Acoustic microbubbles can be used as probes for cellular phenotyping because they can convert ultrasound energy into mechanical cues at the cell–fluid interface while simultaneously reporting the cell’s mechanical response. Depending on how the microbubble is configured relative to the cell, two main mechanisms can be exploited: (i) tethered microbubbles, where ligand-functionalized bubbles are bound to specific membrane receptors (e.g., integrins, sialoglycans) and transmit radiation forces or secondary Bjerknes forces directly into the cytoskeleton and (ii) adjacent or embedded microbubbles, where bubble oscillations drive strong acoustic streaming that deforms suspended cells through localized shear and extensional flows. In both cases, the bubble and cell motion encode effective mechanical properties such as stiffness, viscoelasticity, and deformability, enabling mechanical phenotyping that can be correlated with cell state, lineage, or disease.

The acoustic tweezing cytometry (ATC) framework established a canonical implementation of tethered microbubble mechanophenotyping. Fan et al. functionalized lipid microbubbles with RGD peptides to bind integrins on adherent cells and applied pulsed ultrasound fields to generate lateral radiation forces on individual bubbles [107]. The resulting submicron bubble displacements, tracked by high-speed microscopy, were interpreted within a viscoelastic model of the bubble–cell system to quantify cytoskeletal contractility, creep compliance, and recovery dynamics. Chen et al. extended this concept to two-bubble acoustic tweezing cytometry (TB-ATC), in which a pair of integrin-bound microbubbles attached to the same cell is driven such that their scattered fields interact, producing secondary Bjerknes forces that generate controlled stretching and compression across the cell cortex [108]. This two-bubble configuration allows spatially patterned loading and reveals local mechanical heterogeneities that cannot be captured by single-point indentation techniques. More recently, mechanical models have linked ATC-induced displacements to active contractility changes and viscoelastic remodeling, providing a quantitative basis for using tethered microbubbles as calibrated mechanophenotyping probes at the single-cell level [109,110].

**Figure 5:**
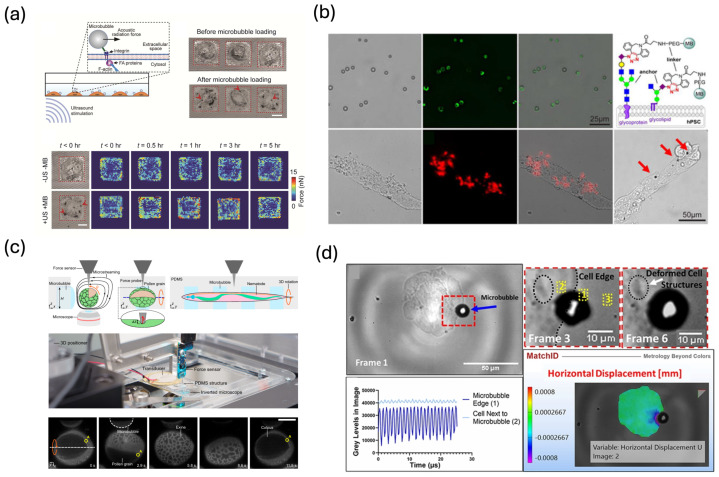
(**a**) Acoustic tweezing cytometry (ATC) modulates cytoskeletal contractility of human mesenchymal stem cells. Scale bar, 30 μm. Reprinted with permission from reference [111]. (**b**) DBCO-functionalized microbubbles target metabolically labeled sialoglycans on hPSCs, enabling molecularly specific acoustic interrogation of cell-surface glycans. Reprinted with permission from reference [114]. (**c**) Acoustically excited microbubble-induced deformation of a single tumor cell, providing a local probe of single-cell mechanical properties. Scale bar: 50 μm. Reprinted with permission from reference [119]. (**d**) Acoustic microbubble setup for 3D manipulation and micro-indentation, allowing controlled force application and mechanical sensing in three-dimensional cell cultures. Reprinted with permission from reference [116].

Acoustic bubble sensing has been applied in studying stem-cell mechanobiology. Xue et al. used cyclic ATC loading via integrin-targeted microbubbles on human mesenchymal stem cells (hMSCs) and showed that repeated mechanical stimulation increases cytoskeletal tension, promotes YAP/TAZ nuclear localization, and enhances osteogenic differentiation while suppressing adipogenic pathways (Figure 5a) [111]. In human embryonic stem cells (hESCs), Topal and co-workers demonstrated that localized ATC actuation at colony edges rapidly reorganizes actin, modulates E-cadherin–mediated junctions, and triggers early differentiation markers, effectively coupling subcellular mechanical perturbation to morphogenesis and fate specification [112]. Fan et al. further used integrin-bound microbubbles to mechanically phenotype hESCs during differentiation, correlating bubble-based viscoelastic metrics with the emergence of lineage-specific gene expression [113]. Building on these studies, Li et al. engineered dibenzocyclooctyne (DBCO)-functionalized microbubbles that specifically attach to metabolically labeled sialoglycans on human pluripotent stem cells (hPSCs) and then used ultrasound to displace these bubbles and read out receptor mechanics from their motion (Figure 5b) [114]. By comparing the displacement and recovery of sialoglycan-anchored versus integrin-anchored microbubbles, they showed that different cell-surface receptors on hPSCs have distinct mechanical properties and elicit different mechanoresponses during early differentiation, demonstrating acoustic bubble sensing as a receptor-specific mechanophenotyping tool for stem cells.

Acoustic microbubble mechanophenotyping has also been applied to immune and cancer cells to reveal disease-relevant mechanical signatures. Hong et al. employed ATC to compare macrophages containing intracellular clofazimine drug crystals, macrophages exposed to soluble drug, and cells that had internalized inert beads [115]. Analysis of microbubble creep and recovery revealed that crystal-laden macrophages became softer and more elastic, whereas bead-loaded or soluble-drug–treated cells exhibited increased stiffness. By increasing ultrasound amplitude and pulse duration, the same microbubble–cell constructs were used to mobilize intracellular crystals, mechanically disrupting the host cells in a process termed mechanopharmaceutical cytotripsy. This work shows how the same acoustic bubble platform can provide both mechanical phenotyping and targeted mechanical intervention. Pattinson et al. developed a high-speed acoustic device to quantify the dynamics of tumor cell–microbubble interaction, enabling precise measurement of how oscillating contrast microbubbles deform and displace nearby tumor cells in vitro (Figure 5d) [116]. The resulting deformation patterns and relaxation kinetics can be used to differentiate cell lines with distinct cytoskeletal architectures or invasive potentials, pointing to microbubble-driven deformation as a functional marker of malignancy. Complementary in vitro and in vivo studies using ultrasound-stimulated microbubbles have shown that controlled acoustic actuation can suppress aggressive phenotypes, enhance radiosensitivity, and remodel the tumor microenvironment, with associated changes in cell morphology, adhesion, and viability that reflect underlying mechanical vulnerabilities [117,118].

Beyond static or adherent cell systems, several studies have applied acoustic bubble phenotyping to microfluidic environments, where bubble oscillations generate localized streaming flows that deform cells suspended in flow. Xie et al. developed a microfluidic deformability assay in which a microscale bubble is generated via optothermal heating near target cells in a chamber and then acoustically actuated to produce strong local acoustic streaming [78]. The resulting shear and extensional flows deform nearby cells, and quantitative analysis of cell shape changes under known streaming conditions yields effective deformability for each cell as a mechanical biomarker. Using this platform, they measured the mean deformability of tens of HeLa, HEK, and HUVEC cells in a single experiment, distinguished their mechanical properties, and showed that cytochalasin treatment increased HeLa deformability by disrupting the actin cortex. Embedded or side-wall microbubbles in microfluidic devices have also been employed to manipulate and probe single cells through bubble-driven streaming and radiation forces. In these architectures, gas cavities are patterned into the channel walls; with acoustic actuation, the trapped bubbles oscillate and generate strong vortical streaming and localized radiation-force landscapes that can trap, rotate, and translate cells with high precision (Figure 5c) [15]. By coupling such embedded-bubble manipulation with independent force probes, researchers have reconstructed three-dimensional mechanical properties of cells and small organisms, demonstrating that acoustic bubble platforms can be integrated with other microfluidic and micromechanical tools for comprehensive phenotyping [119]. More broadly, these microfluidic implementations highlight a distinct but complementary mechanism to tethered microbubbles: instead of direct receptor-mediated loading, cells experience controlled hydrodynamic stresses generated by bubble oscillations, enabling mechanophenotyping of suspended or weakly adherent cells that are difficult to probe using conventional contact methods.

Theoretical and modeling studies have provided a mechanistic foundation for these diverse experimental configurations. Maksymov et al. developed analytical and numerical models showing how gas-bubble oscillation modes are modified by the viscoelastic properties of adjacent cells and tissues, demonstrating that changes in cortical tension, cytoskeletal stiffness, or membrane viscosity can shift resonance frequencies, alter harmonic content, and modulate radiation-force-induced displacements [68]. Zinin and Allen further introduced a shell-model framework for biological cells in the acoustic field of an oscillating bubble, showing that cell-wall deformation is governed by the surface area modulus and the viscosities of the internal and external fluids, with a characteristic frequency at which area strain is maximal and can be sufficient to rupture small, low-quality-factor *Q* bacteria [120]. Combined with cell-scale models of ATC and streaming flows [109], these analyses clarify how measurable bubble dynamics encode cell mechanical properties across tethered, adjacent, and embedded geometries.

### 3.4. Thrombotic and Vascular Diagnostic Sensing

**Figure 6:**
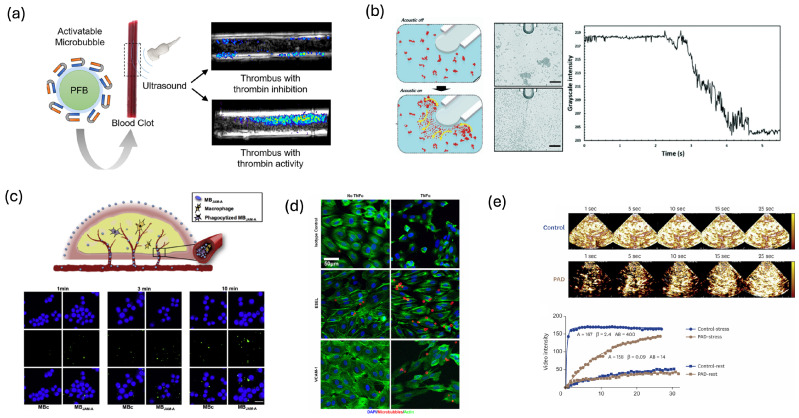
(**a**) Thrombin-activatable microbubbles for ultrasound detection of clot activity. Reprinted with permission from reference [122]. (**b**) Acoustic bubble-mediated thrombolysis in a microfluidic channel, with image-based quantitative analysis of thrombolytic efficiency. Scale bar: 80 μm. Reprinted with permission from reference [125]. (**c**) Targeted microbubbles interacting with activated macrophages and schematic illustration of the process how of JAM-A microbubbles preferentially recognizing vulnerable plaques. Reprinted with permission from reference [126]. (**d**) Microbubble binding on resting versus inflammatory endothelium, serving as noninvasive acoustic indicators of endothelial activation. Reprinted with permission from reference [127]. (**e**) Contrast-enhanced perfusion imaging in peripheral artery disease, using microbubble signal as an acoustic marker for microvascular blood flow and tissue perfusion. Reprinted with permission from reference [128].

Targeted acoustic microbubbles have been widely developed as molecular and functional sensors for thrombotic and vascular pathologies. A major thrust has focused on thrombus-targeted sensing. Nakatsuka et al. developed fibrin-targeted microbubbles that bind specifically to non-occlusive clots and enable in vivo visualization of otherwise undetectable thrombi [121]. Because these microbubbles accumulate on fresh fibrin, the resulting enhancement and persistence of backscatter provide a functional readout of clot presence and activity, with the potential to distinguish acute from chronic thrombi. Lux et al. introduced thrombin-activatable microbubbles whose shells incorporate thrombin-cleavable peptides (Figure 6a) [122]. In regions of active coagulation, enzymatic cleavage converts the agent from an “acoustically silent” to a “bright” state. In this design, the appearance of subharmonic and harmonic signals from previously quiescent microbubbles directly indicates thrombin activity. Wang et al. further advanced the concept with thrombus-targeted theranostic microbubbles that both sense clot burden and deliver fibrinolytic therapy, enabling concurrent ultrasound diagnosis and bleeding-free fibrinolysis [123]. Related work with polymerized-shell fibrin-targeted microbubbles has shown that shell stiffness and binding affinity can be tuned to improve molecular specificity and quantitative assessment of clot composition [124]. These studies demonstrate that acoustic microbubbles can be applied as mechanistically specific sensors of coagulation processes rather than as generic intravascular tracers.

Microfluidic and in vitro models provide controlled platforms for using acoustic bubbles as local sensors of thrombus response to ultrasound. Using a microfluidic sonothrombolysis platform, Zheng et al. systematically evaluated the effects of ultrasound power, thrombolytic agent concentration, and microbubble dose on the size distribution and morphology of thrombus debris for arterial thrombi [129]. Their results show that, in acute arterial sonothrombolysis, ultrasound power has a significant impact on debris size, thereby revealing a clear relationship between acoustic exposure and the safety of sonothrombolysis. Gao et al. designed a microfluidic channel in which bubbles are trapped at defined microcavity sites adjacent to a blood clot; when driven by ultrasound, these bubbles undergo vigorous oscillations and generate microstreaming that shears and fragments the thrombus within a few milliseconds (Figure 6b) [125]. In this work, acoustic bubble-mediated thrombolysis is quantitatively evaluated using image-processing-based sensing metrics: (1) the change in average grayscale intensity within a defined region of interest (ROI), which decreases as large aggregates are broken into smaller fragments or single cells, and (2) the area of the microstreaming vortices around the bubble, extracted from processed images. Together, these ultrasound thrombolysis platforms demonstrate how acoustic bubble-based microfluidic devices can function not only as treatment mimics but also as quantitative sensing tools for probing thrombolytic efficiency and embolic risk.

Beyond thrombi, acoustic bubbles have been extensively employed as sensors of vascular inflammation and endothelial activation. Kaufmann et al. demonstrated that microbubbles targeted to vascular cell adhesion molecule-1 (VCAM-1) can noninvasively map inflammatory activity in atherosclerotic plaques, with the contrast signal correlating with histological measures of VCAM-1 expression [130]. Lindner and others have reviewed how microbubbles directed to VCAM-1, ICAM-1, selectins, and other adhesion molecules report on endothelial activation in early atherosclerosis and post-ischemic injury [131]. More recently, Punjabi et al. used nanobody-based VCAM-1 microbubbles that recognize both murine and human VCAM-1, enabling highly specific ultrasound molecular imaging of vascular inflammation with translational potential [132]. Additional targets such as junctional adhesion molecule-A (JAM-A) have been exploited to sense tight-junction disruption and endothelial barrier dysfunction during vascular inflammation (Figure 6c) [126], while multimodal microbubbles bearing ligands for both endothelial cells and macrophages have been used to track the evolution of inflammatory lesions and monitor response to anti-inflammatory therapy (Figure 6d) [127]. Vascular bubble sensing has also been applied to monitor therapeutic efficacy. Khanicheh et al. showed that VCAM-1–targeted microbubble signal decreases in response to statin therapy in early atherosclerosis, demonstrating that targeted contrast-enhanced ultrasound can serve as a noninvasive readout of endothelial anti-inflammatory treatment [133].

Acoustic bubbles have also been used for contrast-enhanced ultrasound perfusion imaging to sense microvascular flow and reserve in the heart, skeletal muscle, and peripheral vasculature. Wei et al. established a foundational methodology in which continuous infusion of microbubbles, followed by high-power destruction and analysis of replenishment curves, allows quantification of myocardial blood flow from the rate and plateau of microbubble re-entry into the microcirculation [134]. Porter and colleagues translated these principles to clinical myocardial perfusion imaging, where microbubble destruction–reperfusion dynamics are used to detect coronary stenosis and microvascular obstruction [135]. In the peripheral circulation, Duerschmied et al. and Davidson et al. showed that contrast-enhanced ultrasound of the lower extremities can quantify limb perfusion and flow reserve in peripheral arterial disease, using rest–stress changes in microbubble replenishment as sensitive indicators of conduit-vessel stenosis and microvascular dysfunction [136,137]. Nguyen and Davidson further reviewed skeletal muscle CEUS protocols in which microbubble kinetics during exercise or pharmacologic stress provide a dynamic vascular sensor of endothelial function and capillary recruitment (Figure 6e) [128]. Quantitative frameworks, such as those summarized by Greis, use the microbubble signal to directly measure microvascular blood volume and velocity. This approach treats microbubbles as active flow sensors, rather than simple contrast agents used only to visualize anatomy [138].

Acoustic bubble vascular sensing has been extended to specialized vascular beds such as the placenta and hepatic circulation. Wilson et al. highlighted how microbubble contrast-enhanced ultrasound can monitor placental perfusion, intervillous blood flow, and microvascular integrity in pregnancy, positioning microbubbles as minimally invasive sensors of placental physiology and pathophysiology [139]. In the hepatobiliary system, Wen et al. advocated for the implementation of SonoVue microbubbles as intravascular pressure sensors to estimate portal and hepatic venous pressures noninvasively, integrating subharmonic pressure-sensitivity concepts with vascular diagnostic [140].

### 3.5. Pathogen and Infection Sensing

**Figure 7:**
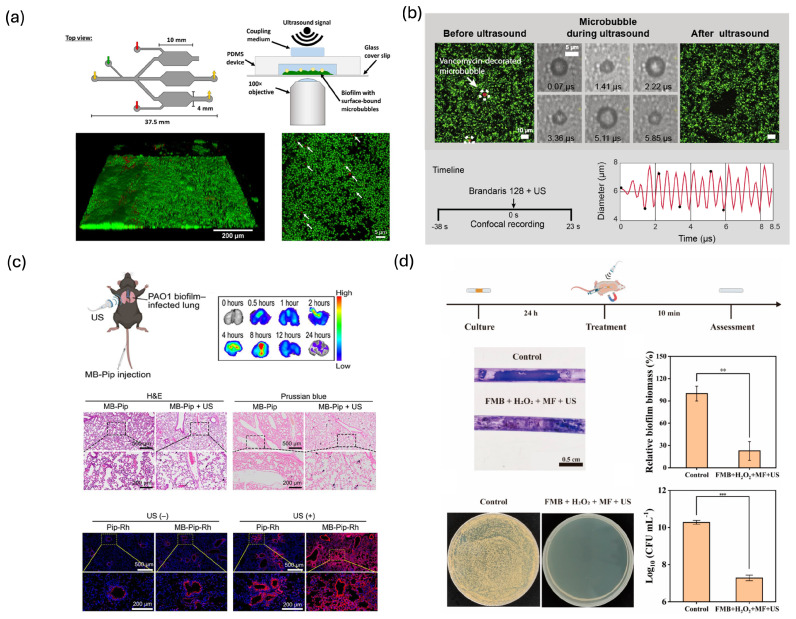
(**a**) Protein-conjugated acoustic bubbles for the selective targeting of *S. aureus* biofilms. White arrows highlight locations of dead cells within the imaging area. Reprinted with permission from reference [141]. (**b**) Microbubble oscillation-induced disruption of bacterial biofilms, where bubble cavitation and backscatter report on biofilm presence and integrity. Reprinted with permission from reference [142]. (**c**) Ultrasound-activated phase-change contrast agents (PCCAs) enhancing antibiotic killing of MRSA biofilms. Reprinted with permission from reference [143]. (**d**) In vivo FMB-microbubble-assisted ultrasound clearing of MRSA catheter biofilms. ** *p* < 0.01, and *** *p* < 0.001. Reprinted with permission from reference [144].

Acoustic microbubbles have been leveraged to sense and interrogate bacterial biofilms and infected tissues, where their cavitation dynamics and targeting specificity provide functional readouts of pathogen burden and treatment response. Protein- or Affimer-functionalized microbubbles have been engineered to bind selectively to *Staphylococcus aureus* biofilms, enabling targeted ultrasound interrogation of biofilm-coated surfaces (Figure 7a) [141]. Upon insonation, these bound microbubbles generate localized cavitation and enhanced backscatter at biofilm regions, allowing ultrasound imaging to map the spatial distribution and mechanical integrity of biofilm structures. Similar ligand-based strategies include vancomycin-decorated microbubbles, in which covalent coupling of vancomycin to the lipid shell allows specific binding to Gram-positive biofilms and provides enhanced contrast and cavitation activity at *S. aureus* infection sites (Figure 7b) [142]. In these systems, the amplitude and persistence of bubble-derived signals serve as proxies for biofilm load and coverage on indwelling devices or tissue surfaces, effectively transforming the microbubble from a passive contrast agent into an active biofilm sensor.

Beyond static imaging, microbubbles facilitate dynamic sensing using ultrasound-targeted microbubble destruction (UTMD) to both disrupt biofilms and sense their susceptibility to antimicrobial treatment. Durham et al. showed that phase-change and microbubble-based contrast agents, when stimulated by ultrasound, enhance antibiotic penetration and killing in methicillin-resistant *S. aureus* (MRSA) biofilms, with changes in acoustic scattering and contrast correlating with biofilm disruption and bacterial killing [145]. Zhao et al. similarly demonstrated that microbubble cavitation can restore the antibiotic susceptibility of *S. aureus* aggregates in synovial fluid, using ultrasound imaging and microbiological assays to link aggregate breakup and viability changes to the cavitation dynamics of the bubbles [146]. More recently, multifunctional catalytic microbubbles incorporating reactive components have been designed to both mechanically disrupt biofilms and catalytically generate reactive oxygen species under ultrasound, with the evolution of the acoustic and chemical signatures reporting on biofilm degradation in chronic lung and implant-associated infections (Figure 7c,d) [143,144,147]. On these platforms, the microbubble behaves as a theranostic agent: its presence and cavitation characteristics provide a real-time acoustic sensor of biofilm structure and treatment efficacy, while simultaneously enhancing bacterial eradication.

### 3.6. Microphysiological Systems and Tissue Microenvironment Sensing

Organ-on-a-chip platforms provide a powerful bridge between conventional cell culture and in vivo models by recreating physiologically relevant architectures, flows, and barrier functions. Within these microfluidic devices, acoustically driven microbubbles act as embedded mechanosensors that report on microvascular permeability, endothelial integrity, and tissue microenvironmental responses to therapy.

**Figure 8:**
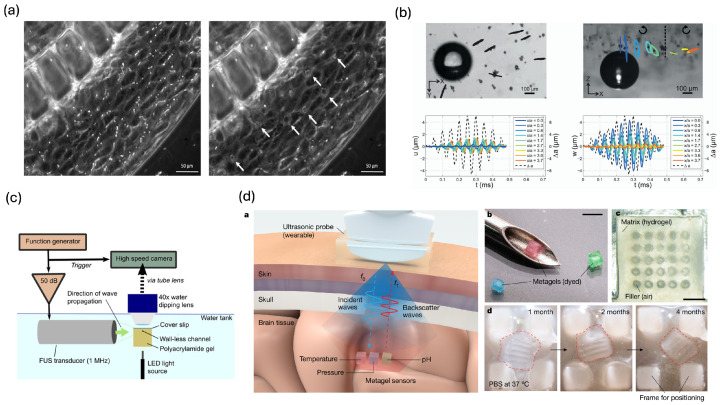
(**a**) Cavitation-induced endothelial intercellular gaps following microbubble exposure, providing a readout of junctional disruption. Before irradiation, MBs is homogeneously-distributed, dispersed particles; Under ultrasound (US) exposure, the acoustic pressure drives MB aggregation, resulting in evenly spaced clusters (white arrow). Reprinted with permission from reference [148]. (**b**) Tracer particle motion around an acoustically driven oscillating microbubble, used to quantify microstreaming flows and local shear. Reprinted with permission from reference [150]. (**c**) Experimental setup for characterizing microbubble oscillation and acoustic streaming in a wall-less polyacrylamide gel channel, enabling sensing of gel-like tissue mechanics. Reprinted with permission from reference [151]. (**d**) The injectable and biodegradable metagels as wireless intracranial physiology sensors using ultrasound reflection. Scale bars, 2 mm (b), 500 μm (c). Reprinted with permission from reference [152].

Acoustic microbubbles have been successfully applied as dynamic point-sensors to interrogate vascular barrier function and mass transport in real time. In endothelialized Microvessel-on-a-Chip models, Park et al. and Grisanti et al. established that the spectral signature of bubble cavitation correlates directly with the magnitude of vascular permeability and therapeutic extravasation, effectively utilizing the bubble as an in situ reporter of transport efficiency (Figure 8a) [74,148]. This sensing capability extends to neurovascular environments, where Conway et al. demonstrated that specific cavitation doses in blood–brain barrier (BBB) models serve as non-invasive surrogate markers for sonoporation-induced opening [149]. By tightly coupling acoustic emissions with physiological readouts—such as transendothelial electrical resistance (TEER) and intracellular calcium dynamics—this study validates the microbubble as a precise, wireless probe for mapping barrier disruption without disturbing the closed microfluidic environment. Complementing these in vitro findings, Hosseinkhah et al. developed a fully coupled bubble–fluid–vessel numerical model showing that transitions in harmonic emissions from capillary-confined bubbles coincide with sharp increases in vessel wall shear and circumferential stresses, thereby quantitatively linking acoustic signatures to BBB opening thresholds [75].

Beyond barrier function, several studies have demonstrated that single microbubbles can act as local, contactless probes of soft tissue mechanics. Tinguely et al. utilized an acoustic bubble in contact with a hydrogel surface to launch Rayleigh-type surface waves. By directly visualizing the resulting deformation field via high-speed microscopy, they showed that wave speed and elliptical surface trajectories are strongly dependent on the gel’s viscoelastic properties, allowing for the extraction of shear modulus at kilohertz frequencies inaccessible to conventional rheometers (Figure 8b) [150]. Complementing this surface-wave approach, Bezer et al. tracked the indentation of a single contrast microbubble into a wall-less hydrogel channel under a controlled primary acoustic radiation force. By fitting the indentation–relaxation curves to a mechanical model, they estimated both the local stiffness and viscosity of soft tissue–mimicking gels (Figure 8c) [151]. Because progressive matrix stiffening is a hallmark of fibrosis and cardiopulmonary disease, such localized acoustic elastography offers a promising non-invasive method to monitor mechanical changes in lung-on-a-chip and heart-on-a-chip platforms.

Recent work has extended the physical principles of resonant gas inclusions from in vitro chips to fully implantable, tissue-like metamaterial sensors. Tang et al. reported an injectable ultrasonic “metagel” for wireless monitoring of intracranial signals, consisting of a bioresorbable hydrogel phononic crystal containing periodically aligned air columns (Figure 8d) [152]. In this architecture, the air columns function as fixed, lattice-confined “bubbles” whose shape and spacing deform in response to environmental changes. Once implanted, deformation of the metagel caused by pressure, temperature, pH, or fluid flow modifies the lattice geometry, resulting in measurable shifts in the peak frequency of reflected ultrasound. In rat and pig models, this sensor achieved centimetre-scale interrogation depths and multiparametric sensitivity comparable to wired clinical benchmarks, while fully degrading over weeks.

## 4. Conclusions and Future Perspectives

Acoustic bubble sensing leverages the exquisite sensitivity of microbubble oscillations to their local biochemical and biomechanical microenvironment, offering a uniquely remote, multifunctional, and label-free modality for measurement and actuation. In this review, we have connected the fundamental physics of single-bubble and collective oscillations with emerging sensing strategies for pressure, flow, viscosity, stiffness, oxygenation, pH, and molecular binding in vitro and in vivo. Across contrast-enhanced ultrasound imaging, microassays, and microphysiological systems, these platforms demonstrate that a single physical entity—an acoustically driven bubble—can simultaneously act as a contrast agent, a microscale local actuator, and an embedded sensor.

Despite this immense promise, several barriers limit the translation of these techniques from laboratory demonstrations to routine, quantitative deployment. First, quantitative readout remains complicated by nonlinear dynamics, multi-bubble coupling, and bubble–boundary interactions that are difficult to model and calibrate. Second, variability in bubble properties (e.g., polydispersity, shell heterogeneity) and boundary conditions across studies impedes reproducibility and standardization. Third, bubble stability in biological environments remains a critical area for improvement. Microbubbles may dissolve via gas diffusion, deflate, or shed shell material under cyclic driving. Overcoming these limitations requires advances in both agent design and operation: robust shells, stabilized gas cores, anti-fouling surface chemistries, and bubble size control. Finally, clinical translation requires strict control over cavitation regimes and defined operational windows for repeated insonation, particularly for applications near delicate barriers, such as the blood–brain barrier (BBB), or in disease-altered tissues.

Looking forward, several directions could transform acoustic bubble sensing into a generalizable technology platform. First, establishing rigorous calibration frameworks is essential. Coupling high-fidelity multiphysics models with reduced-order descriptors, standardized calibration protocols, and uncertainty quantification will be critical for mapping measured acoustic signatures back to absolute microenvironmental quantities. Second, the field stands to benefit from adaptive and data-driven inference: machine learning-assisted signal processing, autonomous ultrasound parameter optimization, and closed-loop control could enable bubbles to act as “intelligent” agents that adaptively sense and modulate their environment. Third, materials and molecular engineering will likely expand bubble sensing beyond primarily mechanical readouts toward multiplexed biochemical sensing. The integration of bubbles with advanced microphysiological systems and 3D tissue models will enable real-time, non-invasive readouts of local stress, transport, and biochemical gradients, complementing existing optical and electrochemical sensors. Furthermore, multimodal approaches that combine bubbles with optical reporters, nanoparticles, or flexible electronics may significantly enhance sensitivity, spatial resolution, and multiplexing capabilities.

Applications are also broadening beyond conventional contrast-enhanced imaging. Near-term opportunities include hemodynamic pressure assessment and thrombus or hemostasis phenotyping. At smaller scales, bubble-enabled mechanobiology is poised to grow from proof-of-concept single-cell studies toward scalable phenotyping in organoids and engineered tissues. Additional emerging directions include infection and biofilm sensing (capturing changes in viscoelasticity and microstructure), barrier and transport assessment in organ-on-chip systems (monitoring endothelial integrity and interstitial permeability), and implantable acoustic resonators for chronic deep-tissue monitoring. Beyond biomedicine, related acoustic bubble paradigms are increasingly relevant in microfluidic quality control (gas emboli detection), industrial process monitoring (emulsions, foams, fermentation), and underwater acoustics, where engineered cavities serve as tunable resonant inclusions for sensing and wave manipulation.

Overall, realizing the potential of acoustic bubble sensing will require coordinated advances at the intersection of acoustics, materials science, engineering, and clinical ultrasound practice. Addressing stability, calibration, and standardization, while leveraging adaptive control and novel agent designs, could yield a new class of programmable, acoustically actuated microtransducers for quantitative biosensing, image-guided interventions, and the probing of disease microenvironments.

## Data Availability

Not applicable.
